# A Case of Compartment Syndrome Due to Out-of-Hospital Intraosseous Misplacement During Cardiopulmonary Resuscitation

**DOI:** 10.7759/cureus.26228

**Published:** 2022-06-23

**Authors:** Annapoorna Singh, Daulath Singh

**Affiliations:** 1 Internal Medicine, University of Missouri-Kansas City (UMKC) School of Medicine, Kansas City, USA; 2 Internal Medicine/Hematology-Oncology, University of Missouri-Kansas City (UMKC), Kansas City, USA

**Keywords:** intraosseous access, resuscitation, compartment syndrome, cardiac arrest, hypoglycemia

## Abstract

Resuscitation relies heavily on gaining access to the circulatory system. During cardiopulmonary resuscitation (CPR), the biggest, most readily accessible vein that does not impede resuscitation is desired. Intraosseous (IO) access is designated for life-threatening emergencies and is a relatively safe procedure with fewer complications. We describe an intriguing and uncommon consequence of out-of-hospital IO placement: compartment syndrome resulting from the displacement of the IO needle by emergency medical services (EMS) workers in a diabetic woman with hypoglycemia. A few hours later, the patient had swelling, discomfort, and loss of motor and sensory sensations at the IO site, necessitating further examinations. The IO needle had traversed both the anterior and posterior cortices of the tibia and was located in the soft tissues along the posterior portion of the tibia as shown by imaging of the afflicted area. Immediate decompression fasciotomy was performed to preserve the patient's limb.

## Introduction

Establishing circulation is a core part of cardiopulmonary resuscitation. Intraosseous (IO) access is typically intended to be used for critical circumstances when conventional or standard venous access methods are not accomplished promptly. It was first described in 1922 [1] and had been widely used in children for drug administration. The long bones of the body are the preferred site; the IO cannulation is a relatively safe procedure with less than 1% of complication risk [2]. We present a case of compartment syndrome (CS) due to the misplacement of IO in the prehospital setting in a diabetic woman with hypoglycemia.

## Case presentation

**Figure 1:**
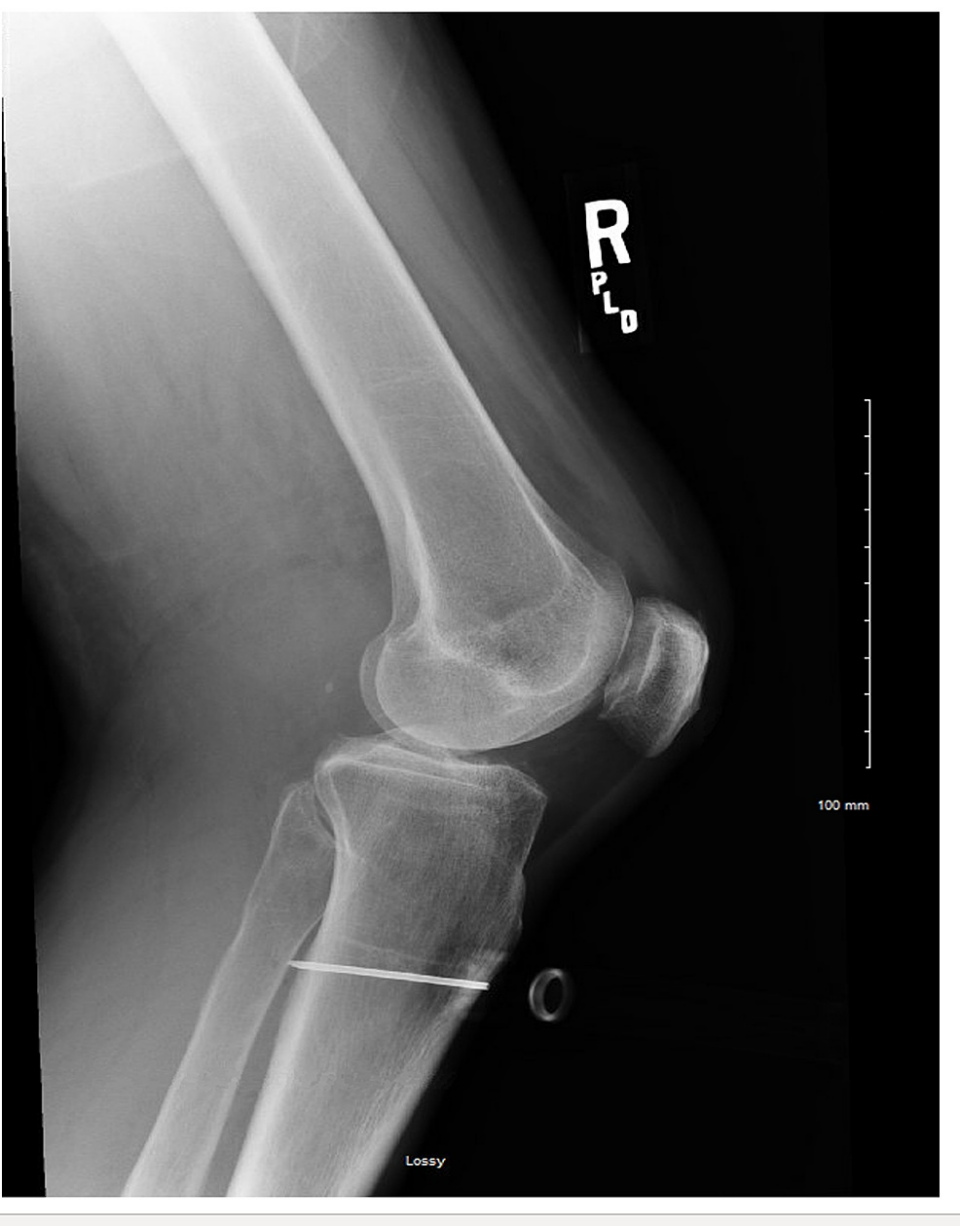
The tip of intraosseous needle is extraosseous, traversing both anterior and posterior cortices along the posterior aspect of the tibia

**Figure 2:**
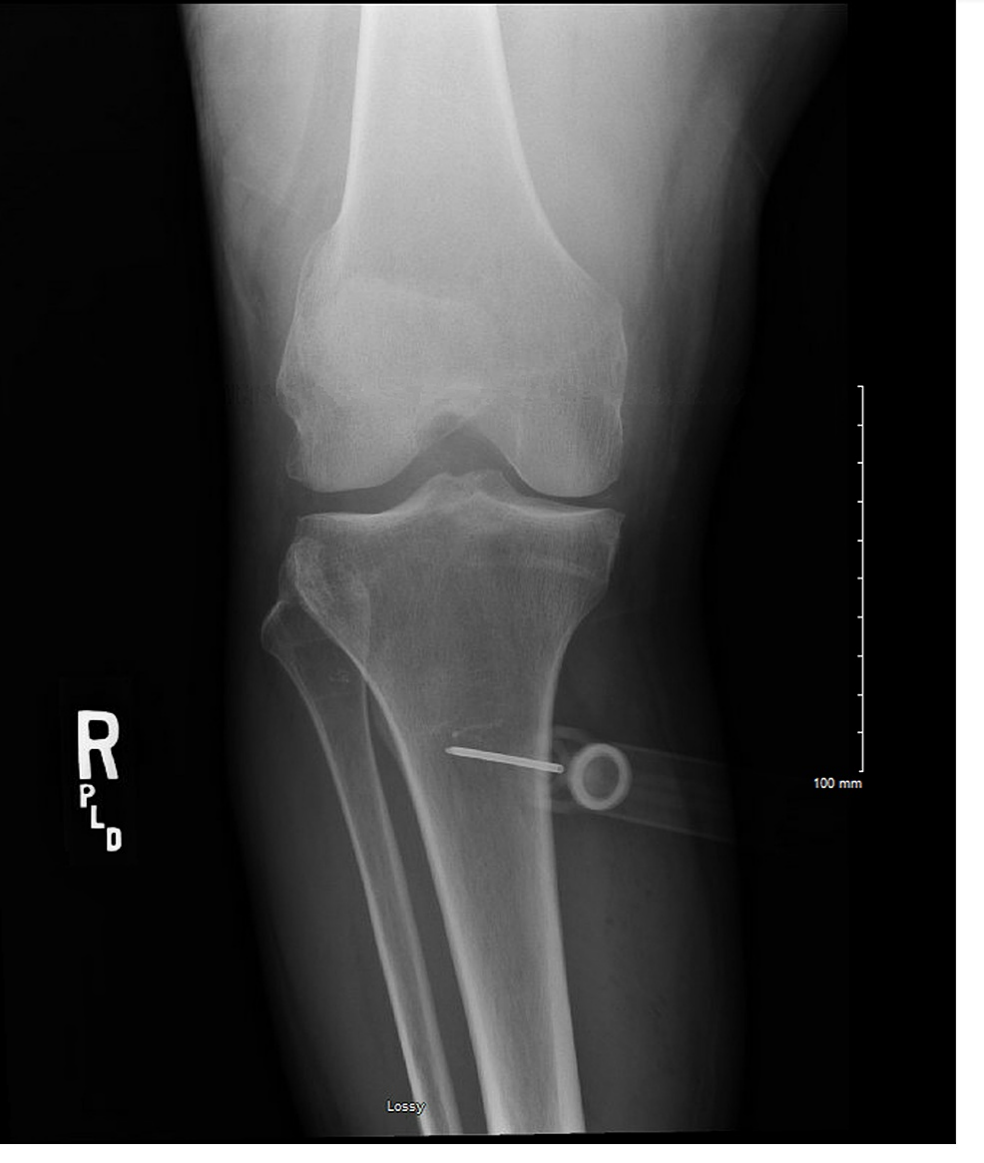
Posteriorly directed intraosseous needle in the proximal tibial diaphysis. The needle tip was extraosseous and was in the soft tissues along the posterior aspect of the tibia.

A 56-year-old woman with a history of diabetes mellitus type-2, decompensated cirrhosis with hepatic encephalopathy, and alcohol use disorder was brought to the hospital via emergency medical services (EMS) acute obtundation due to hypoglycemia. The capillary glucose was <30 mg/dl, and the patient was obtunded with a Glasgow Coma Scale (GCS) score of 3. An intraosseous needle was placed in the field by EMS after several unsuccessful attempts of peripheral IV access (PIV). The patient received dextrose plus glucagon via IO en route to the hospital. On arrival to the emergency department, she was still obtunded with a GCS score of 3. The patient received three dextrose 50% amp infusions with immediate improvement in mental status. Later that day, the patient complained of severe progressive right leg and thigh pain along with swelling. Physical examination showed pain with passive dorsiflexion and plantarflexion of the foot along with diminished sensations and reduced motor strength on the affected side. Radiology of the right knee obtained immediately showed a posteriorly directed IO needle in the proximal tibial diaphysis with the needle extending through both anterior and posterior cortices. The tip of the needle was extraosseous and was in the soft tissues along the posterior aspect of the tibia (Figures 1, 2).

There was also diffuse soft tissue swelling, with a small gas bubble in the medial soft tissues. Orthopedics surgery emergently performed four-compartment fasciotomy decompression for right lower extremity CS with the removal of IO needle with a repeat primary closure on the second day of admission. The patient was later discharged to a skilled nursing facility.

## Discussion

The establishment of access is a critical component of resuscitation. IO infusion is usually reserved for critically ill patients and is possible because of the presence of veins that drain the medullary sinuses in the bone marrow of long bones [3]. Due to their substantial size, these veins are easily accessible during periods of decreased circulation, such as shock. The preferred sites are long bones such as the proximal tibia, femur, distal tibia, proximal humerus, and manubrium. In a trial of 182 adults, tibial IO placement was associated with a significantly higher frequency of initial vascular access success than peripheral IV placement [4]. Any IV medicines and fluids can be administered safely. As with IV treatment, IO infusion has a similar onset of action and serum drug concentrations [5]. Animal studies reveal that the IO route is equally effective as the central intravenous route [6-8] and may be superior to the PIV during cardiac arrest [7]. IO cannulation is generally safe, with fewer than 1% of patients experiencing serious complications [9]. Potential problems include tibial fracture, CS, skin necrosis, osteomyelitis, and subcutaneous abscess. Infusion of fluids into the subcutaneous tissue has caused CS and resulted in the amputation of an extremity [10]. In our case, CS was caused by the displacement of the needle. The appropriate size of the needle should be selected based on the insertion site and the subcutaneous fatty tissue. Medical health personnel should be aware of the clinical signs and symptoms of the CS. In case of doubt, correct placement in the bone marrow cavity can be confirmed radiologically or by bedside Doppler ultrasound.

## Conclusions

In medically urgent circumstances, intravenous infusion allows for the rapid acquisition of vascular access when conventional venous access methods are not possible or cannot be obtained quickly. Obtaining access to circulation is one of the most crucial steps in cardiopulmonary resuscitation, hence should be done promptly without any delays. Thus, IO access is critical as it can be accomplished quickly and is inserted through the long bones of the body into the long non-collapsible veins that retain their tortuosity in the presence of shock/circulatory collapse and are readily accessible.

It is important to aspirate fluid after obtaining IO access to ensure correct placement into the site. Providers should be aware of the clinical presentation of CS following IO placement and should quickly obtain orthopedics consult as early diagnosis and immediate decompression surgery are the keys to avoiding amputation.

## References

[1] (2022). The circulation in the mammalian bone-marrow. https://scholar.google.com/scholar_lookup?journal=Am+J+Physiol&title=The+circulation+of+the+mammalian+bone+marrow&author=CK+Drinker&author=KR+Drinker&author=CC+Lund&volume=62&issue=1&publication_year=1922&pages=1-92&.

[2] Petitpas F, Guenezan J, Vendeuvre T, Scepi M, Oriot D, Mimoz O (2016). Use of intra-osseous access in adults: a systematic review. Crit Care.

[3] Blumberg SM, Gorn M, Crain EF (2008). Intraosseous infusion: a review of methods and novel devices. Pediatr Emerg Care.

[4] Celık T, Ozturk C, Balta S, Demırkol S, Iyısoy A (2016). A new route to life in patients with circulatory shock: intraosseous route. Am J Emerg Med.

[5] 5] E. C. Institute (ECI (2021). Circulation - intraosseous access. Emergency Care Institute (ECI), Oct.

[6] Spivey WH, Lathers CM, Malone DR (1985). Comparison of intraosseous, central, and peripheral routes of sodium bicarbonate administration during CPR in pigs. Ann Emerg Med.

[7] Neufeld JD, Marx JA, Moore EE, Light AI (1993). Comparison of intraosseous, central, and peripheral routes of crystalloid infusion for resuscitation of hemorrhagic shock in a swine model. J Trauma.

[8] Orlowski JP (1994). Emergency alternatives to intravenous access. Intraosseous, intratracheal, sublingual, and other-site drug administration. Pediatr Clin North Am.

[9] Bardes J, Strumwasser A (2018). Techniques of intraosseous access. Atlas of Critical Care Procedures.

[10] Taylor CC, Clarke NM (2011). Amputation and intraosseous access in infants. BMJ.

